# Supplementary material for: ssNMRlib: a comprehensive library and tool box for acquisition of solid-state nuclear magnetic resonance experiments on Bruker spectrometers
Source: Magn Reson (Gott). 2020 Dec 23;1(2):331–45. doi: 10.5194/mr-1-331-2020 (PMC10500710; doi:10.5194/mr-1-331-2020)
Supplement: The supplement related to this article is available online at: https://doi.org/10.5194/mr-1-331-2020-supplement. [file mr-1-331-supplement.pdf]

Supplement of Magn. Reson., 1, 331–345, 2020
https://doi.org/10.5194/mr-1-331-2020-supplement
© Author(s) 2020. This work is distributed under
the Creative Commons Attribution 4.0 License.

Open Access
MAGNETIC RESONANCE

Supplement of

ssNMRlib: a comprehensive library and tool box for acquisition of solid-state nuclear magnetic resonance experiments on Bruker spectrometers

Alicia Vallet et al.

Correspondence to: Alicia Vallet (alicia.vallet@ibs.fr) and Paul Schanda (paul.schanda@ist.ac.at)

The copyright of individual parts of the supplement might differ from the CC BY 4.0 License.

Listing S1 : parameter naming convention in ssNMRlib

General naming conventions for CPs, plw, spw, cnst

CP power levels are entered in kHz and are generally assigned to the three channels as:

30-39 : 13C	H-C CP : 1	Examples :
40-49 : 1H	H-N CP : 2	13C RF field for HC CP → cnst 31
50-56 : 15N	N-CO : 3	1H RF field for HC CP → cnst 41
57-63 : Midpoint ramp	N-CA : 4	

Hard Pulses

if 1H detection

p1 : H 90 degree pulse (us) @ plw1
p2 : C 90 degree pulse (us) @ plw2
p3 : N 90 degree pulse (us) @ plw3
p4 : 2H 90 degree pulse (us) @plw4

if 13C detection

p1 : C 90 degree pulse (us) @ plw1
p2 : H 90 degree pulse (us) @ plw2
p3 : N 90 degree pulse (us) @ plw3
p4 : 2H 90 degree pulse (us) @ plw4

if 15N detection

p1 : N 90 degree pulse (us) @ plw1
p2 : H 90 degree pulse (us) @ plw2
p3 : C 90 degree pulse (us) @ plw3

CP constants

HN CP

cnst 42 : RF field on 1H (kHz)
cnst 52 : RF field on 15N (kHz)
cnst 62 : Midpoint of the ramp
spnam 42 : File name for the ramp
p 45 : CP duration (us)

HC/HCA/HCO CP

cnst 31 : RF field on 13C (kHz)
cnst 41 : RF field on 1H (kHz)
cnst 61 : Midpoint of the ramp
spnam 41 : File name for the ramp
p 43 : CP duration (us)

N-CO CP

cnst 33 : RF field on 13C (kHz)
cnst 43 : RF field on 1H for decoupling (kHz)
cnst 53 : RF field on 15N (kHz)
cnst 63 : Midpoint of the ramp
spnam 53 : File name for the ramp
p 53 : CP duration (us)

N-CA CP

cnst 34 : RF field on 13C (kHz)
cnst 44 : RF field on 1H for decoupling
cnst 54 : RF field on 15N (kHz)
cnst 60 : Midpoint of the ramp
spnam 54 : File name for the ramp
p 35 : CP duration (us)

Simultaneous HCN CP

cnst 31 : RF field on 13C (kHz)
cnst 41 : RF field on 1H (kHz)
cnst 51 : RF field on 15N (kHz)
cnst 61 : Midpoint of the ramp
spnam 41 : File name for the ramp
p 43 : CP duration (us)

HA-CA CP

cnst 35 : RF field on 13C (kHz)
cnst 45 : RF field on 1H (kHz)
cnst 57 : Midpoint of the ramp
spnam 45 : File name for the ramp
p 34 : CP duration (us)

INEPT

d 13 : INEPT delay HC (sec)
d 14 : INEPT delay HN (sec)
d 15 : INPET delay CA-CB (sec)
d 16 : INEPT delay CO-CA, CO transverse (sec)
d 17 : INEPT delay CO-CA, CA transverse (sec)
d 53 : INEPT delay N-CO (sec)
d 54 : INEPT delay N-CA (sec)

Transfer 13C-13C

13C RFDR

cnst 20 : Spinning rate (Hz)
cnst 37 : 13C offset during RFDR
d 8 : Mixing time
p 37 : RFDR duration (us)

DARR

cnst 5 : RF field on 1H (kHz)
cnst 20 : Spinning rate (Hz)
d 5 : Mixing time (ms)
d 22 : Rotation period

DREAM CA-CB

cnst 6 : RF field on 1H (kHz)
cnst 7 : RF of the DREAM (kHz)
cnst 17 : 13C offset for DREAM
cnst 59 : Midpoint of the ramp
p 17 : DREAM duration (us)
spnam 7 : DREAM ramp

CP BSH CO-CA

cnst 38 : RF field for the CO-CA (kHz)
cnst 58 : Midpoint of the ramp
p 38 : CP duration (us)
p 39 : Trim duration before BSH (us)
p 40 : Trim duration after BSH (us)
spnam 38 : BSH-CP ramp

TOCSY / DIPSI

cnst 15 : RF field on 13C (kHz)
d 15 : Mixing duration (sec)
cpdprg 8 : Mixing sequence
pcpd 5 : Pulse length for the mixing sequence

Alfresco

p 50 : Alfresco chirp pulse duration (us)
cnst 50 : Alfresco RF power (kHz)
spnam 49 : chirp pulse file name
spnam 50 : chirp pulse file name
l4 : mixing loop

Transfer 1H-1H

Bass-SD

cnst 48 : 1H carrier during Bass-SD
cnst 49 : RF Bass-SD at midpoint (kHz)
cnst 59 : Midpoint of the Bass-SD shape
spnam 49 : Bass-SD element
p 49 : Bass-SD duration (us)
d 8 : z-filter delay (sec)

1H RFDR

cnst 20 : Spinning rate (Hz)
cnst 37 : 1H offset during RFDR
d 8 : Mixing time (sec)
p 37 : RFDR duration (us)
L0 : RFDR loop

Z-mix

d 8 : Z-mix delay (sec)

Transfer 15N-13C

TEDOR

L1 : Loop number for TEDOR experiment

Transfer 15N-15N

PDSD

d 5 : PDSD mixing time (sec)

Dynamics

REDOR
p 18 : 1H pulse 180° (us)
p 19 : 13C pulse 180° (us)
cnst 20 : Spinning rate (Hz)
d 7 : Redor shift delay (sec)
L0=0 : Redor loop (2 rotor period min)
L1=0 : Reference

R1rho
cnst 25 : R1rho field strength (kHz)
p 18 : heat compensation pulse
p 25 : spinlock
p 26 : 13C/15N degree pulse (us)
d 24 : R1rho max delay

T2
d 25 : T2 delay

T1
d 25 : T1 delay (sec)

CEST
cnst 17 : 1H decoupling (kHz) @ plw17
cnst 27 : 15N RF field (Hz)
cnst 29 : Set to 0 for reference experiment
p 17 : Decoupling pulse length (us)
d 31 : CEST delay (sec)
cpdprg 5 : 1H decoupling sequence
cpdprg 6 : CEST 15N sequence

EXSY
d 31 : EXSY mixing time (sec)

Selective 13C pulses

spnam 10 : EBURP2 on-resonance CO	according to p11 for the pulse duration
spnam 11 : EBURP2-TR on-resonance CO	according to p12 for the pulse duration
spnam 13 : ISNOB 2 off-resonance CO	according to p13 for the pulse duration
spnam 14 : REBURP on-resonance CA	according to p14 for the pulse duration
spnam 15 : REBURP on-resonance CA-CB	according to p15 for the pulse duration
spnam 16 : EBURP2 off-resonance CO	according to p16 for the pulse duration
spnam 17 : EBUR2P on-resonance CA	according to p17 for the pulse duration
spnam 18 : ISNOB2 off-resonance CA	according to p18 for the pulse duration
spnam 19 : REBURP on-resonance CO	according to p19 for the pulse duration

Decoupling

13C decoupling during 1H detection
cpdprg 1 : sequence used for the 13C decoupling during 1H detection
pcpd 1 : pulse length in decoupling sequence (us)
cnst 11 : 13C decoupling power (kHz) @ plw11

1H decoupling during 13C/15N evolution/detection
cpdprg 2 : sequence used for the 1H decoupling during evolution/detection time
pcpd 2 : pulse length in decoupling sequence (us)
cnst 12 : 1H decoupling power (kHz) @ plw12

15N decoupling during 1H detection
cpdprg 3 : sequence used for the 15N decoupling during 1H detection
pcpd 3 : pulse length in decoupling sequence (us)
cnst 13 : 15N decoupling power (kHz) @ plw13

2H decoupling

cpdprg 5 : sequence used for the 15N decoupling during 1H detection
pcpd 5 : pulse length in decoupling sequence (us)
cnst 14 : 2H decoupling power (kHz) @ plw14

Solvent suppression

cnst 24 : 1H RF field for solvent suppression (kHz)
d 30 : duration for the solvent suppression (sec)
cpdprg 4 : decoupling sequence
pcpd 7 : pulse length for the solvent suppression (us)

Other constants

cnst 1 : 13C reference RF field (kHz), from p 21 (for calculation)
cnst 2 : 1H reference RF field (kHz), from p 22 (for calculation)
cnst 3 : 15N reference RF field (kHz), from p 23 (for calculation)
cnst 4 : 2H reference RF field (kHz), from p 24 (for calculation)
cnst 10 : Multiplier for acqt=cnst10*1u set to 1
cnst 15 : Center of CB (39 ppm)
cnst 16 : Center of CO (175 ppm)
cnst 17 : Center of CA (54 ppm)
cnst 18 : Carrier between CA and CO (100 ppm)
cnst 20 : Spinning rate (Hz)

Other delays

d 20 : rotor period (calculated from cnst 20)
d 21 : half rotor-period

Listing S2 : currently implemented experiments

NMRlib tools

- **Template maker** → Implement the current experiment to the library
- **Export** →Export the loaded template to a folder for sharing
- **Import** → Import an experiment from your forlder to your library
- **Delete** → Delete the loaded template from the library

Set-up experiments

- KBr
- Adamantane

Set pulse lengths → Set 90° pulse widths

Security → Control your experiment before to launch it

Remove → Remove all the best ramp in order to start a new optimization or user default constants
Recap file → Create or charge a recap file of your optimizations
Load your contants → Load selectively transfer parameters
Save your pulse program → Save your pulse program inside your experiment (not compiled version)

Proteins

- **Proton detection**

    - **Set user default parameters: Water suppression parameters, D1 & RG**

    - **Calibrations**
        - **O1 Calibration**

        - **Hard Pulse**
            - 1H Calibration based on hNH CP experiment
            - 1H Calibration based on hCH CP experiment
            - 15N Calibration based on hNH CP experiment
            - 13C Calibration based on hCH CP experiment
            - 1H Direct calibration
            - 1H Calibration based on hNH INEPT experiment
            - 1H Calibration based on hCH INEPT experiment
            - 15N Calibration based on hNH INEPT experiment
            - 13C Calibration based on hNH INEPT experiment

        - **Cross Polarization**
            - CP HN optimization
            - CP HC optimization
            - CP HCO optimization
            - CP HCA optimization
            - CP HACA optimization

CP NCO optimization
CP NCA optimization
CP simultaneous hCH hNH optimization
BSH CO-CA optimization

INEPT
hNH
hCH
INEPT delay COCA, CO transverse
INEPT delay COCA, CA transverse

1D spectra
Basic 1D experiment
1H R1

Hetero 2Ds HN/HC CP/INEPT
Experiments without 2H decoupling
hNH CP
hNH refINEPT
hCH CP
hCH refINEPT
Simultaneous CH and NH CP
Experiments with 2H decoupling
hCH CP
hCH refINEPT
Simultaneous CH and NH CP

Backbone assignment
3D Experiments
Experiments without 2H decoupling
hCOcaNH → HN(i)-N(i)-CO(i)
Scalar CO-ca
Scalar CO-ca semi constant-time
Dipolar CO-ca
hCONH → HN(i)-N(i)-CO(i-1)
hCANH → HN(i)-N(i)-CA(i)
hcoCAcoNH → HN(i)-N(i)-CA(i-1)
hCAcoNH → HN(i)-N(i)-CA(i-1)
Dipolar CA-co
hcaCBcaNH → HN(i)-N(i)-CB(i)
hcaCBcacoNH → HN(i)-N(i)-CB(i-1)
Scalar ca-co
Dipolar ca-co
hNcocaNH → HN(i)-N(i)-N(i+1)
Scalar co-ca
Dipolar co-ca
hNcacoNH → HN(i)-N(i)-N(i-1)
Dipolar ca-co

Experiments with 2H decoupling

- **hCOcaNH** → HN(i)-N(i)-CO(i)
  - Scalar CO-ca
- **hcaCBcaNH** → HN(i)-N(i)-CB(i)
- **hcaCBcacoNH** → HN(i)-N(i)-CB(i-1)

4D Experiments

Experiments without 2H decoupling

- **hCOCANH** → HN(i)-N(i)-CO(i)-CA(i)
  - Scalar CO-CA
  - Scalar CO-CA semi contant-time
  - Scalar CO-CA with double semi constant-time
  - Dipolar CO-CA
- **hCOCAcoNH** → HN(i)-N(i)-CO(i-1)-CA(i-1)
  - Scalar CO-CA
  - Scalar CO-CA-co semi constant-time
- **hCACONH** → HN(i)-N(i)-CA(i-1)-CO(i-1)
  - Dipolar CA-CO
- **hcaCBcaCONH** → HN(i)-N(i)-CB(i-1)-CO(i-1)
  - Scalar ca-CO
  - Scalar ca-CO semi constant-time
  - Dipolar ca-CO
- **hcaCBCANH** → HN(i)-N(i)-CA(i)-CB(i)
- **hcaCBCAcoNH** → CB(i-1)-CA(i-1)-N(i)-HN(i)
  - Scalar CA-co
  - Scalar CA-co semi constant-time
- **hCONCAHA** → CO(i-1)-N(i)-CA(i)-HA(i)
- **hNCOcaNH** → N(i+1)-CO(i)-N(i)-HN(i)
  - Scalar CO-ca
  - Dipolar CO-ca
- **hNcoCANH** → N(i+1)-CA(i)-N(i)-HN(i)
  - Scalar co-CA
  - Dipolar co-CA
- **HNcocaNH** → HN(i+1)-N(i+1)-N(i)-HN(i)
  - Scalar co-ca

Experiments with 2H decoupling

- **hCOCANH** → HN(i)-N(i)-CO(i)-CA(i)
- **hcaCBcaCONH** → HN(i)-N(i)-CB(i-1)-CO(i-1)
- **hcaCBCANH** → HN(i)-N(i)-CA(i)-CB(i)
- **hcaCBCAcoNH** → CB(i-1)-CA(i-1)-N(i)-HN(i)
- **hNCOcaNH** → N(i+1)-CO(i)-N(i)-HN(i)
- **hNcoCANH** → N(i+1)-CA(i)-N(i)-HN(i)

Side-chain assignment

Experiments without 2H decoupling

- hCCH TOCSY
- hCCH refINEPT TOCSY

hCCH refINEPT

Experiment with 2H decoupling

hCCH refINEPT

Dynamics

Experiments without deuterium decoupling

Dipolar couplings

- hCH REDOR
- hNH REDOR
- hCH INEPT REDOR
- hNH INEPT REDOR

Longitudinal Relaxation

- R1 hCH 13C → CP
- R1 hCONH 13CO → CP
- R1 hCANH 15N → CP
- R1 hNH 15N → CP
- R1 hCH 13C → INEPT
- T1 hCH 1H → CP
- T1 hCH 1H → INEPT

Transverse Relaxation

- R1rho hCH 1H → CP
- R1rho hCH 13C → CP
- R1rho hCONH 13CO → CP
- R1rho hCANH 15N → CP
- R1rho hNH 15N → CP
- T2 hNH 1H → CP
- T2 hCH 1H → CP

Transverse Relaxation

- hCH EXSY → CP
- hNH EXSY → CP
- Simultaneous hNH hCH EXSY → CP
- CEST hNH → CP

Experiments with deuterium decoupling

- hCH EXSY → CP
- Simultaneous hCH hNH EXSY → CP
- REDOR hCH → CP
- R1rho hCH → CP

Distance Measurements

Experiments with deuterium decoupling

- HhNH HHRFDR
- Simultaneous h(C/N)hh(C/N)H HHRFDR
- Simultaneous Hh(C/N)H HHRFDR
- Bass-SD HhCH

HhCONH Hzmix
HhCANH Hzmix

Experiments with deuterium decoupling
Simultaneous h(C/N)hh(C/N)H HHRFDR
Simultaneous Hh(C/N)H HHRFDR

Carbon detection

Set user default parameters: D1 & RG

Calibrations
Hard Pulse
13C Calibration based on hC CP experiment
1H Calibration based on hC CP experiment
13C Direct calibration

Cross Polarization
CP HC optimization
CP HCO optimization
CP HCA optimization
CP NCO optimization
CP NCA optimization

INEPT
hC
NCO INEPT
NCA INEPT
CC INEPT

C-C transfer
CC INEPT
DREAM
DARR CO-CX duration
DARR CA-CO duration
RFDR CO-CX duration
RFDR CA-CO duration
CO-CA BSH CP
CA-CO BSH CP

1D Experiments
13C direct
CP HC
INEPT HC
hNCA → double CP
hNCO → double CP

1D Relaxation Experiments

1H T1 → via CP or INEPT
1H T2 → via CP or INEPT
13C T1 → via CP
13CA T2 → via CP
13CO T2 → via CP

2D Experiments

CC experiments

hCC → CP DARR
hCC → CP DREAM
hCC → CP RFDR
hCC → CP Alfresco
hCOCA → CP BSH
hChhC → CP HHmixing
hCC → CP INEPT
CC → Direct INEPT
hCC → INEPT

HC experiments

HC HETCOR → CP
HC HETCOR → INEPT

NC experiments

hNhhC → CP HHmixing
hNCA → Double CP
hNCO → Double CP
hNCA → INEPT
hNCO → INEPT
hNcoCA → CP BSH
hNcoCA → INEPT
hNcaCO → CP BSH
hNcaCO → INEPT

3D Experiments

hCANCO → CP /CP /CP
hCONCA → CP /CP /CP
hNCACB → CP /CP /DREAM
hCONcaCB → CP/CP/CP/DREAM
hNCACO → CP/CP/BSH
hNCOCA → CP/CP/BSH
hNCOCX → CP /CP /DARR
hNCACX → CP /CP /DARR
hNCOCX → CP /CP /RFDR
hNCACX → CP /CP /RFDR
hCOCACB → CP/BSH/DREAM

4D Experiments

hCANCOCX → CP /CP /CP/DARR
hCONCACX → CP /CP /CP/DARR
hCONCACB → CP/CP/CP/DREAM

Nitrogen detection

Set user default parameters: D1 & RG

Calibrations

Hard Pulse

15N Calibration based on hN CP experiment
15N Direct calibration

Cross Polarization

CP HN optimization

INEPT

hN

1D

HN → CP or INEPT
15N T1 → CP
15N T2 → CP

2D

HN HETCOR → CP or INEPT
hNN → CP PDSD
hNhhN → CP

Listing S3 : Example of parameter file (“recap file”), which can be saved (and retrieved) from the ssNMRlib window

```
# System: Avance III HD 600 NMR spectrometer

 MAS Probe used : B6292_00230 (PH MASDVT 600S3 TL2 CNDH 1.3mm)
 Probe diameter: 1.3 mm
 Spinning rate: 55.006 (kHz)
 Temperature : 258.0 (K) with Gas Flow measured : 1300.000000 (lph)
 Topspin data location : /home/avallet/nmrsolids/2020-06-22_PythonTest_13mm/2
 Sample id in sample database : 516

 1Hcal  Hard_Pulse_on_1H : 3.28 (us)    @ 2020-06-24 10:04:05
 1H_CPdefcal  Hard_Pulse_for_CP_on_1H : 3.28 (us)   @ 2020-06-24 10:04:05
 13Ccal  Hard_Pulse_on_13C : 2.85 (us)    @ 2020-06-24 10:07:09
 13C_CPdefcal  Hard_Pulse_for_CP_on_13C : 2.85 (us)   @ 2020-06-24 10:07:09
 15Ncal  Hard_Pulse_on_15N : 3.40 (us)   @ 2020-06-24 10:10:45
 15N_CPdefcal  Hard_Pulse_for_CP_on_15N : 3.40 (us)   @ 2020-06-24 10:10:45


 O1_calibration  O1p : 4560 (Hz)   @ 2020-06-24 10:03:58


 Water_Suppression_Delay  D 30 : 0.15 (sec)   @ 2020-06-24 10:11:07
 RFfield_for_Water_Suppression  CNST 24 : 12 (kHz)   @ 2020-06-24 10:11:07


 CP_HN_BestRamp   Ramp used: ramp90100.100   @ 2020-06-24 10:18:05
 TargetField_1H_CP_HN_ramp90100_cal   CNST 42 : 84 (kHz)   @ 2020-06-24 10:12:05
 TargetField_15N_CP_HN_ramp90100_cal   CNST 52 : 35.001 (kHz)   @ 2020-06-24 10:14:21
 CP_HN_duration    P 45 : 800 (us)   @ 2020-06-24 10:16:35


 CP_HCA_BestRamp   Ramp used: ramp50100.100   @ 2020-06-24 10:23:45
 CP_HCA_duration    P 43 : 3000 (us)   @ 2020-06-24 10:18:32
 TargetField_1H_CP_HCA_ramp50100_cal   CNST 41 : 14.454545 (kHz)   @ 2020-06-24 10:20:05
 TargetField_13C_CP_HCA_ramp50100_cal   CNST 31 : 39.998 (kHz)   @ 2020-06-24 10:22:25


 CP_HCO_BestRamp   Ramp used: ramp70100.100   @ 2020-06-24 10:28:26
 TargetField_1H_CP_HCO_ramp70100_cal   CNST 41 : 84.000000 (kHz)   @ 2020-06-24 10:23:58
 CP_HCO_duration    P 43 : 4750.000000 (us)   @ 2020-06-24 10:25:35
 TargetField_13C_CP_HCO_ramp70100_cal   CNST 31 : 35.002 (kHz)   @ 2020-06-24 10:27:05


 CP_CN_BestRamp   Ramp used: ramp90100.100   @ 2020-06-24 10:33:00
 TargetField_15N_CP_CN_ramp90100_cal   CNST 53 : 36.363636 (kHz)   @ 2020-06-24 10:28:48
 CP_CN_duration    P 53 : 9368.421053 (us)   @ 2020-06-24 10:30:02
 TargetField_13C_CP_CN_ramp90100_cal   CNST 33 : 16.999 (kHz)   @ 2020-06-24 10:31:31


 CP_NCA_BestRamp   Ramp used: ramp90100.100   @ 2020-06-24 10:39:14
 TargetField_15N_CP_NCA_ramp90100_cal   CNST 54 : 38 (kHz)   @ 2020-06-24 10:33:17
 CP_NCA_duration    P 35 : 9000.0 (us)   @ 2020-06-24 10:35:09
 TargetField_13C_CP_NCA_ramp90100_cal   CNST 34 : 15.362636 (kHz)   @ 2020-06-24 10:37:28

 INEPT_delay_HN   D 14 : 0.001 (sec)  @ 2020-06-24 10:42:08
```

INEPT_delay_HC   D 13 : 0.001 (sec) @ 2020-06-24 10:43:35

INEPT_delay_NCO   D 53 : 0.010 (sec) @ 2020-06-24 10:45:45

INEPT_delay_NCA   D 54 : 0.01 (sec) @ 2020-06-24 10:47:27

TargetField_BSH_CP_cal   CNST 38 : 21.741217 (kHz)   @ 2020-06-24 10:50:01

TargetField_BSH_CP_duration_cal   P 38 : 4500 (us)   @ 2020-06-24 10:51:52

DARR_COCX_duration   D 5 : 0.2 (sec)   @ 2020-06-24 10:54:02

CP_DREAM_duration   P 17 : 5000 (us)   @ 2020-06-24 10:56:05
TargetField_DREAM   CNST 7 : 6.103091 (kHz)   @ 2020-06-24 10:57:58

RFDR_COCX_duration   D 8 : 0.004 (sec)   @ 2020-06-24 10:59:24

Listing S4 : Security checks – How does it work?

Each pulse sequence within the solid-state NMRlib module is associated with a jython script named as “experiment_p.py”. This script allows the user to define selectively which parameters will be verified for this specific experiment.
In order to perform the safety checks, the safety script will compare : the parameter value within the topspin experiment with the safety table adapted by the user for his probes.
This safety table, so-called “Security_table_SSNMRlib.txt”, is located in : “/NMRlib/py” and contains all the maximum values authorized for each probe.
An extract of this file is shown below.

SIZE	PULSE_TYPE	ELEMENT	DEFINITION	CNST_NAME	MAX_kHz	PULSE	DURATION	UNIT	SPNAM	MIDPOINT_RAMP	SEQ_DETECTION
0.7	HP	1H	1H_HP	-	-	P1	15	us	-	-	1H
0.7	HP	1H	1H_HP	-	-	P2	15	us	-	-	13C
0.7	HP	1H	1H_HP	-	-	P2	15	us	-	-	15N
0.7	HP	13C	13C_HP	-	-	P1	15	us	-	-	13C
0.7	HP	13C	13C_HP	-	-	P2	15	us	-	-	1H
0.7	HP	13C	13C_HP	-	-	P3	15	us	-	-	15N
0.7	HP	15N	15N_HP	-	-	P1	15	us	-	-	15N
0.7	HP	15N	15N_HP	-	-	P3	15	us	-	-	13C
0.7	HP	15N	15N_HP	-	-	P3	15	us	-	-	1H
0.7	HP	2H	2H_HP	-	-	P4	25	us	-	-	1H
0.7	HP	2H	2H_HP	-	-	P4	25	us	-	-	13C
0.7	HP	2H	2H_HP	-	-	P4	25	us	-	-	15N
0.7	HP	13C	13C_HP_for_CP	-	-	P21	5	us	-	-	1H
0.7	HP	13C	13C_HP_for_CP	-	-	P21	5	us	-	-	13C
0.7	HP	13C	13C_HP_for_CP	-	-	P21	5	us	-	-	15N
0.7	HP	1H	1H_HP_for_CP	-	-	P22	4	us	-	-	1H
0.7	HP	1H	1H_HP_for_CP	-	-	P22	4	us	-	-	13C
0.7	HP	1H	1H_HP_for_CP	-	-	P22	4	us	-	-	15N
0.7	HP	15N	15N_HP_for_CP	-	-	P23	6	us	-	-	1H
0.7	HP	15N	15N_HP_for_CP	-	-	P23	6	us	-	-	13C
0.7	HP	15N	15N_HP_for_CP	-	-	P23	6	us	-	-	15N
0.7	HP	2H	2H_HP_for_CP	-	-	P24	25	us	-	-	1H
0.7	HP	2H	2H_HP_for_CP	-	-	P24	25	us	-	-	13C
0.7	HP	2H	2H_HP_for_CP	-	-	P24	25	us	-	-	15N
0.7	CP	HN	RF_Field_1H	CNST42	190	P45	6000	us	42	62	-
0.7	CP	HN	RF_Field_15N	CNST52	55	P45	6000	us	-	-	-
0.7	CP	HC	RF_Field_13C	CNST31	70	P43	6000	us	-	-	-
0.7	CP	HC	RF_Field_1H	CNST41	190	P43	6000	us	41	61	-
0.7	CP	HCA	RF_Field_13C	CNST31	70	P43	6000	us	-	-	-
0.7	CP	HCA	RF_Field_1H	CNST41	190	P43	6000	us	41	61	-
0.7	CP	HCO	RF_Field_13C	CNST31	70	P43	6000	us	-	-	-
0.7	CP	HCO	RF_Field_1H	CNST41	190	P43	6000	us	41	61	-
0.7	CP	HACA	RF_Field_13CA	CNST35	70	P34	7000	us	-	-	-
0.7	CP	HACA	RF_Field_1HA	CNST45	190	P34	7000	us	45	57	-
0.7	CP	NCO	RF_Field_13CO	CNST33	70	P53	10000	us	-	-	-
0.7	CP	NCO	RF_Field_15N	CNST53	55	P53	10000	us	53	63	-
0.7	CP	NCA	RF_Field_13CA	CNST34	70	P35	10000	us	-	-	-
0.7	CP	NCA	RF_Field_15N	CNST54	55	P35	10000	us	54	60	-
0.7	CP	sim_HCN	RF_Field_13C	CNST31	70	P43	6000	us	-	-	-
0.7	CP	sim_HCN	RF_Field_1H	CNST41	190	P43	6000	us	41	61	-
0.7	CP	sim_HCN	RF_Field_15N	CNST51	55	P43	6000	us	-	-	-

The security check is automatically performed when an experiment is loaded from NMRlib. Moreover, if the user wants to change a parameter inside topspin, a “security button” can be hit, at any time, in order to re-do all the checks.
If a problem appears, a pop-up is generated showing the actual problematic topspin value and the specification in order to alert the user. This warning is also written in the topspin terminal.

```
############ Security checking #############
PULSE PROGRAM : av_hNH_cp_cp_miss.IBS
Security table : /home/avallet/NMRlib/py/Security_table_SSNMRlib.txt
P21, P22, P23 & P24 are used for the power calculation
Hard Pulse P1 for the nucleus 1H:   2.75 us, spec:   15.00 us: Ok.
Hard Pulse P2 for the nucleus 13C:   2.93 us, spec:   15.00 us: Ok.
Hard Pulse P3 for the nucleus 15N:   3.80 us, spec:   15.00 us: Ok.
Hard Pulse P21 for the nucleus 13C:   2.93 us, spec:    6.00 us: Ok.
Hard Pulse P22 for the nucleus 1H:   2.75 us, spec:    5.00 us: Ok.
Hard Pulse P23 for the nucleus 15N:   3.80 us, spec:    7.00 us: Ok.
D1 :   0.89 sec, spec:    0.60 sec: Ok
CP HN CNST 42:  15.00 kHz, spec:  120.00 kHz: Ok
CP HN duration P  45: 1000.00 us, spec:  6000.00 us: Ok
CP HN SPNAM 42 ramp50100.100 :  max RF on CNST 42:  18.75 kHz, max spec: 120.00 kHz : Ok
WARNING CP HN CNST 52:  51.00 kHz, spec:   50.00 kHz
Water suppression CNST24:  10.00 kHz, spec:   30.00 kHz: Ok
CP duration D30 for water suppression:   0.16 sec, spec:    0.20 sec: Ok
Decoupling CNST 12:   10.00 kHz, Max spec: 120.00 kHz: Ok
CNST 12: Decoupling time during indirect dimension F 1:  0.0263 sec, Max spec: 0.0300 sec: Ok
Decoupling CNST 13:    5.00 kHz, Max spec:  30.00 kHz: Ok
Decoupling on CNST 13 during the aquisition:  0.0150 sec, Max spec: 0.0300 sec: Ok
```

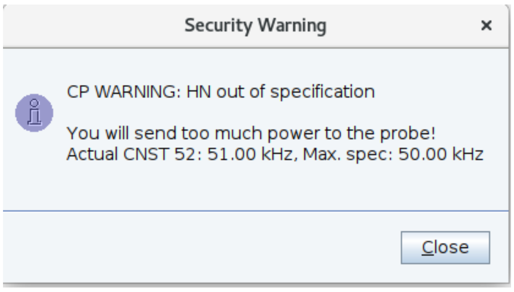

In this way, each pulse sequence has its own personalized security which is adapted for its use and to the probe specification :

For each pulse sequence :

- All the parameters (water suppression elements, pulse, duration & RF power) within the pulse sequence are checked according to the probe.
- In a ramped RF shape, for CP or DREAM/BSH, the maximum value of the ramp is taken into account. To this end, the maximum value in the shape file is extracted, and the corresponding kHz value is calculated.
- The safety check verifies that midpoint of the ramp that is stored in a constant (e.g. cnst62 = 95 for a 90-to-100 ramp) and used for calculating the power level of the ramp, is indeed the mean of that ramp. This is done by explicitly verifying the ramp in the shape file.
- In CP experiments, the values of the two corresponding RF fields is checked. If the values are too far from Hartmann-Hahn conditions a popup window informs the user. This may point to mis-calibration of the reference pulses, as the CP power levels are calculated from them.
- If a list is used in the pulse sequence (e.g. the spin-lock duration in an $R_{1\rho}$ experiment), the safety check retrieves all the values within this list and determines the maximal value. This ensures that all values are within the safety limits.
- If an element is repeated multiple times, the safety check will verify the total duration, i.e. the duration of the unit element times the number of repeats.
- The decoupling times are retrieved automatically for the whole direct and indirect dimensions, i.e. the maximum evolution time (t_{1max}) is considered.

In addition, for the calibrations :

- As the parameter optimization array is define within the python script, negative values are automatically discarded.
- In a parameter optimization, the safety check verifies that none of the experiments of the array exceeds the allowed safety limits.
- Clicking the button of a calibration experiment generally starts the acquisition automatically. However, if a safety problem is detected, the calibration is not launched automatically.

Listing S4 : Security checks – How does it work?

Each pulse sequence within the solid-state NMRlib module is associated with a jython script named as “experiment_p.py”. This script allows the user to define selectively which parameters will be verified for this specific experiment.
In order to perform the safety checks, the safety script will compare : the parameter value within the topspin experiment with the safety table adapted by the user for his probes.
This safety table, so-called “Security_table_SSNMRlib.txt”, is located in : “/NMRlib/py” and contains all the maximum values authorized for each probe.
An extract of this file is shown below.

SIZE	PULSE_TYPE	ELEMENT	DEFINITION	CNST_NAME	MAX_kHz	PULSE	DURATION	UNIT	SPNAM	MIDPOINT_RAMP	SEQ_DETECTION
0.7	HP	1H	1H_HP	-	-	P1	15	us	-	-	1H
0.7	HP	1H	1H_HP	-	-	P2	15	us	-	-	13C
0.7	HP	1H	1H_HP	-	-	P2	15	us	-	-	15N
0.7	HP	13C	13C_HP	-	-	P1	15	us	-	-	13C
0.7	HP	13C	13C_HP	-	-	P2	15	us	-	-	1H
0.7	HP	13C	13C_HP	-	-	P3	15	us	-	-	15N
0.7	HP	15N	15N_HP	-	-	P1	15	us	-	-	15N
0.7	HP	15N	15N_HP	-	-	P3	15	us	-	-	13C
0.7	HP	15N	15N_HP	-	-	P3	15	us	-	-	1H
0.7	HP	2H	2H_HP	-	-	P4	25	us	-	-	1H
0.7	HP	2H	2H_HP	-	-	P4	25	us	-	-	13C
0.7	HP	2H	2H_HP	-	-	P4	25	us	-	-	15N
0.7	HP	13C	13C_HP_for_CP	-	-	P21	5	us	-	-	1H
0.7	HP	13C	13C_HP_for_CP	-	-	P21	5	us	-	-	13C
0.7	HP	13C	13C_HP_for_CP	-	-	P21	5	us	-	-	15N
0.7	HP	1H	1H_HP_for_CP	-	-	P22	4	us	-	-	1H
0.7	HP	1H	1H_HP_for_CP	-	-	P22	4	us	-	-	13C
0.7	HP	1H	1H_HP_for_CP	-	-	P22	4	us	-	-	15N
0.7	HP	15N	15N_HP_for_CP	-	-	P23	6	us	-	-	1H
0.7	HP	15N	15N_HP_for_CP	-	-	P23	6	us	-	-	13C
0.7	HP	15N	15N_HP_for_CP	-	-	P23	6	us	-	-	15N
0.7	HP	2H	2H_HP_for_CP	-	-	P24	25	us	-	-	1H
0.7	HP	2H	2H_HP_for_CP	-	-	P24	25	us	-	-	13C
0.7	HP	2H	2H_HP_for_CP	-	-	P24	25	us	-	-	15N
0.7	CP	HN	RF_Field_1H	CNST42	190	P45	6000	us	42	62	-
0.7	CP	HN	RF_Field_15N	CNST52	55	P45	6000	us	-	-	-
0.7	CP	HC	RF_Field_13C	CNST31	70	P43	6000	us	-	-	-
0.7	CP	HC	RF_Field_1H	CNST41	190	P43	6000	us	41	61	-
0.7	CP	HCA	RF_Field_13C	CNST31	70	P43	6000	us	-	-	-
0.7	CP	HCA	RF_Field_1H	CNST41	190	P43	6000	us	41	61	-
0.7	CP	HCO	RF_Field_13C	CNST31	70	P43	6000	us	-	-	-
0.7	CP	HCO	RF_Field_1H	CNST41	190	P43	6000	us	41	61	-
0.7	CP	HACA	RF_Field_13CA	CNST35	70	P34	7000	us	-	-	-
0.7	CP	HACA	RF_Field_1HA	CNST45	190	P34	7000	us	45	57	-
0.7	CP	NCO	RF_Field_13CO	CNST33	70	P53	10000	us	-	-	-
0.7	CP	NCO	RF_Field_15N	CNST53	55	P53	10000	us	53	63	-
0.7	CP	NCA	RF_Field_13CA	CNST34	70	P35	10000	us	-	-	-
0.7	CP	NCA	RF_Field_15N	CNST54	55	P35	10000	us	54	60	-
0.7	CP	sim_HCN	RF_Field_13C	CNST31	70	P43	6000	us	-	-	-
0.7	CP	sim_HCN	RF_Field_1H	CNST41	190	P43	6000	us	41	61	-
0.7	CP	sim_HCN	RF_Field_15N	CNST51	55	P43	6000	us	-	-	-

The security check is automatically performed when an experiment is loaded from NMRlib. Moreover, if the user wants to change a parameter inside topspin, a “security button” can be hit, at any time, in order to re-do all the checks.
If a problem appears, a pop-up is generated showing the actual problematic topspin value and the specification in order to alert the user. This warning is also written in the topspin terminal.

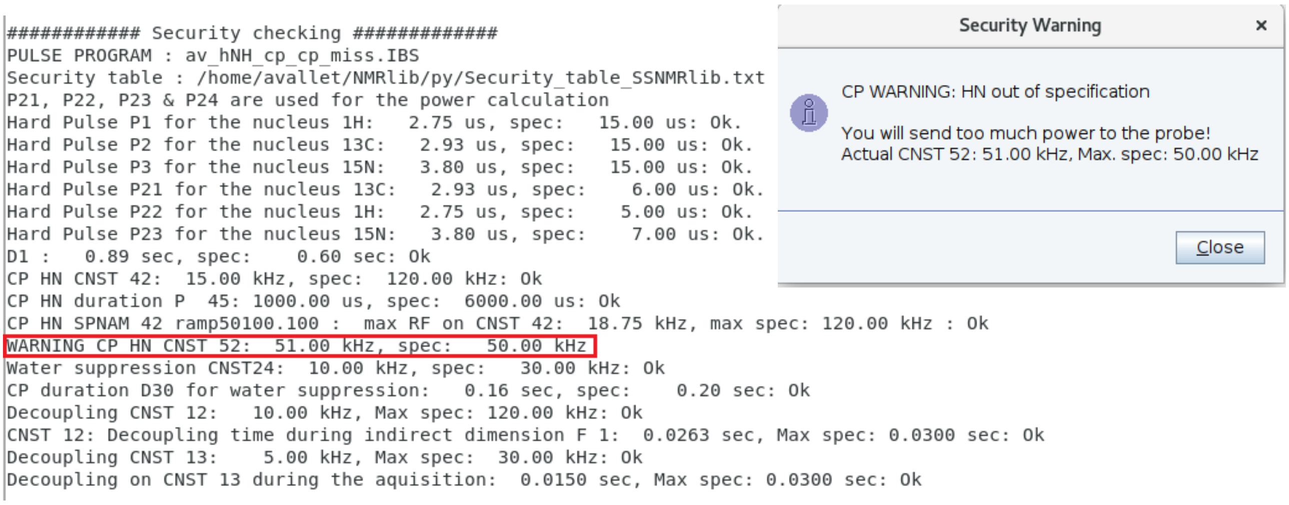

In this way, each pulse sequence has its own personalized security which is adapted for its use and to the probe specification :

For each pulse sequence :

- All the parameters (water suppression elements, pulse, duration & RF power) within the pulse sequence are checked according to the probe.
- In a ramped RF shape, for CP or DREAM/BSH, the maximum value of the ramp is taken into account. To this end, the maximum value in the shape file is extracted, and the corresponding kHz value is calculated.
- The safety check verifies that midpoint of the ramp that is stored in a constant (e.g. cnst62 = 95 for a 90-to-100 ramp) and used for calculating the power level of the ramp, is indeed the mean of that ramp. This is done by explicitly verifying the ramp in the shape file.
- In CP experiments, the values of the two corresponding RF fields is checked. If the values are too far from Hartmann-Hahn conditions a popup window informs the user. This may point to mis-calibration of the reference pulses, as the CP power levels are calculated from them.
- If a list is used in the pulse sequence (e.g. the spin-lock duration in an $R_{1\rho}$ experiment), the safety check retrieves all the values within this list and determines the maximal value. This ensures that all values are within the safety limits.
- If an element is repeated multiple times, the safety check will verify the total duration, i.e. the duration of the unit element times the number of repeats.
- The decoupling times are retrieved automatically for the whole direct and indirect dimensions, i.e. the maximum evolution time (t_{1max}) is considered.

In addition, for the calibrations :

- As the parameter optimization array is define within the python script, negative values are automatically discarded.
- In a parameter optimization, the safety check verifies that none of the experiments of the array exceeds the allowed safety limits.
- Clicking the button of a calibration experiment generally starts the acquisition automatically. However, if a safety problem is detected, the calibration is not launched automatically.

Listing S5 : Adding an experiment in NMRlib

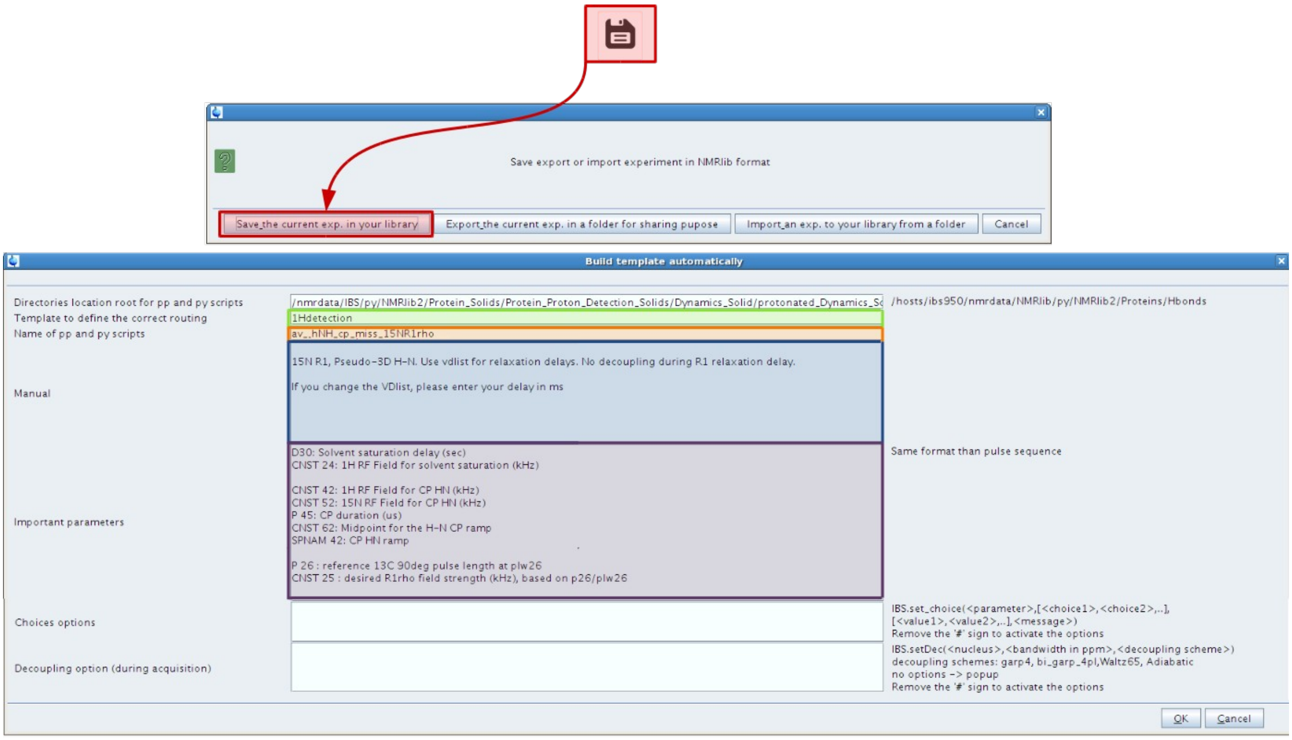

Routing file :

if 1H detection experiment -> 1Hdetection
if 13C detection experiment -> hCONCaCx4D
if 15N detection experiment -> 15Ndetection

Experiment name

Comments :

Experiment name, reference

Important parameters :

Parameters that can be popup for checking during the set-up of the experiment
